# Noninvasive Prediction of Sperm Retrieval Using Diffusion Tensor Imaging in Patients with Nonobstructive Azoospermia

**DOI:** 10.3390/jimaging9090182

**Published:** 2023-09-08

**Authors:** Sikang Gao, Jun Yang, Dong Chen, Xiangde Min, Chanyuan Fan, Peipei Zhang, Qiuxia Wang, Zhen Li, Wei Cai

**Affiliations:** 1 Department of Radiology, Tongji Hospital, Tongji Medical College, Huazhong University of Science and Technology, Wuhan 430030, China; gaosikang@tjh.tjmu.edu.cn (S.G.); minxiangde0129@126.com (X.M.); fanchanyuan@tjh.tjmu.edu.cn (C.F.); zhangpeipei337@126.com (P.Z.); guaiqiuqiu1981@163.com (Q.W.); zhenli@hust.edu.cn (Z.L.); 2 Department of Urology, Tongji Hospital, Tongji Medical College, Huazhong University of Science and Technology, Wuhan, 430030, China; tjmuyj@tjh.tjmu.edu.cn; 3 Department of Pathology, Tongji Hospital, Tongji Medical College, Huazhong University of Science and Technology, Wuhan, 430030, China; chendong2266@tjh.tjmu.edu.cn

**Keywords:** nonobstructive azoospermia, diffusion tensor imaging, microdissection testicular sperm extraction, mean diffusivity, fractional anisotropy, sperm retrieval rate

## Abstract

Microdissection testicular sperm extraction (mTESE) is the first-line treatment plan for nonobstructive azoospermia (NOA). However, studies reported that the overall sperm retrieval rate (SRR) was 43% to 63% among men with NOA, implying that nearly half of the patients fail sperm retrieval. This study aimed to evaluate the diagnostic performance of parameters derived from diffusion tensor imaging (DTI) in predicting SRR in patients with NOA. Seventy patients diagnosed with NOA were enrolled and classified into two groups based on the outcome of sperm retrieval during mTESE: success (29 patients) and failure (41 patients). Scrotal magnetic resonance imaging was performed, and the DTI parameters, including mean diffusivity and fractional anisotropy, were analyzed between groups. The results showed that there was a significant difference in mean diffusivity values between the two groups, and the area under the curve for mean diffusivity was calculated as 0.865, with a sensitivity of 72.2% and a specificity of 97.5%. No statistically significant difference was observed in fractional anisotropy values and sex hormone levels between the two groups. This study demonstrated that the mean diffusivity value might serve as a useful noninvasive imaging marker for predicting the SRR of NOA patients undergoing mTESE.

## 1. Introduction

Male infertility may be responsible for 40–60% of cases where couples are unable to conceive [1]. Azoospermia is the major cause and most serious type of male infertility, affecting around 1% of the male population and up to 15% of infertile men [2]. Azoospermia can be classified into two types: obstructive azoospermia and nonobstructive azoospermia (NOA). NOA affects approximately 60% of azoospermic patients and has been considered to be the most severe manifestation of male infertility [3]. NOA refers to the dysfunction of testicular spermatogenesis, in which no sperm or only a small amount of sperm are produced, resulting in the absence of sperm in semen. NOA can be divided into three categories: congenital NOA (chromosome structure and number abnormalities, Y chromosome microdeletion, cryptorchidism, etc.), acquired NOA (infection, tumor, trauma, drugs and toxins, radiation, etc.), and idiopathic NOA.

More than half of NOA patients have minute foci of spermatogenesis, which is the basis of the treatment of NOA patients by surgical sperm retrieval [4]. Microdissection testicular sperm extraction (mTESE) is the first-line treatment plan for NOA and has a high sperm retrieval rate (SRR) and a low incidence of complications [5]. However, studies have shown that the overall sperm retrieval rate (SRR) for men with NOA undergoing mTESE ranged from 43% to 63%, indicating that nearly half of the patients failed to retrieve sperm [6,7]. Additionally, traditional predictors such as age, testicular volume, follicle-stimulating hormone, testosterone, or inhibin B have proven unreliable in determining SRR [8,9,10,11,12,13,14]. A recent study reported that the SRR of NOA patients could be improved by locating the best perfusion area of the testis with ultrasound [15]. However, the outcomes of ultrasound were highly operator-dependent and less repeatable. There is currently a lack of efficient and noninvasive methods for predicting SRR before mTESE. Finding a noninvasive method to predict SRR and avoid unnecessary mTESE has received widespread attention.

Compared to ultrasound, magnetic resonance imaging (MRI) has higher tissue contrast and repeatability. As a noninvasive examination, functional MRI has shown good diagnostic accuracy in the qualitative and quantitative diagnosis of testicular diseases [16,17,18,19]. Diffusion tensor imaging (DTI) is a functional MRI technique that demonstrates the direction and speed of water molecule diffusion in biological tissues [20]. DTI can provide both mean diffusivity (MD) and fractional anisotropy (FA) values, which might reflect the structure and organization of complex tissues such as the brain, heart, prostate, kidney, liver, and breast [21,22,23,24]. Recent studies have reported that the quantitative parameter values of DTI are useful to differentiate the testes of NOA patients from controls and the testes of infertile men with varicocele from controls [18,25]. Consequently, DTI holds potential as a tool for predicting SRR in NOA patients prior to mTESE.

In this study, we aimed to investigate the ability of quantitative DTI parameters to assess the SRR in patients with NOA.

## 2. Materials and Methods

### 2.1. Study Population

The current study protocol was approved by the Ethics Committee of Tongji Hospital, Tongji Medical College, Huazhong University of Science and Technology, and all participants gave written informed consent. From September 2017 until March 2023, we conducted a study involving 70 patients (mean age, 30 years; age range, 24–40 years) diagnosed with NOA at our hospital. The inclusion criteria were as follows: absence of sperm confirmed by high-power centrifugation of two separate semen samples; and pathological results after mTESE suggested dysfunction of spermatogenesis. The exclusion criteria were as follows: obstructive factors confirmed in the sperm outflow channel; patients with retrograde ejaculation (diagnosis was made by the detection of large numbers of motile spermatozoa and fructose by the first post-ejaculation urinalysis); presence of lesions in the testis; Y chromosome microdeletions of the AZFa or AZFb [26]; untreated patients with idiopathic hypogonadotropic hypogonadism (sperm could be found in semen after hormone therapy in patients with idiopathic hypogonadism) [27]; and age over 40 years [28]. A scrotal MRI was performed within 1 month prior to mTESE or testicular sperm aspiration.

### 2.2. MRI Protocol

All patients were scanned on a 3.0 T MRI scanner (MAGNETOM Skyra, Siemens Healthcare, Erlangen, Germany) with an 18-channel body coil and a 32-channel spine coil. The scrotal MRI was performed with patients in a supine position with their feet advanced. The parameters of the axial T2-weighted fast spin–echo sequence were as follows: repetition time/echo time: 6500/104 ms; layer thickness: 3 mm; layer spacing: 0 mm; field of view: 180 × 180 mm^2^; matrix: 384 × 320; and total time: 3 min 16 s. The parameters of the axial DTI fat-saturated single-shot spin–echo planar were as follows: repetition time/echo time: 4900/89 ms; layer thickness: 3 mm; layer spacing: 0 mm; field of view: 300 × 240 mm^2^; matrix: 140 × 112; 20 diffusion directions; b-value: 0, 800 s/mm^2^; and total time: 4 min 10 s.

### 2.3. Image Interpretation and Data Analysis

The DTI model equation is as follows:

$$\mathrm{MD}=\mathrm{ADC}=\lambda_{m}=\frac{\left(\lambda_{1}+\lambda_{2}+\lambda_{3}\right)}{3}$$

$$\mathrm{FA}=\sqrt{\frac{3}{2}}\;.\sqrt{\frac{{\left(\lambda_{1}-\lambda_{m}\right)}^{2}+{\left(\lambda_{2}-\lambda_{m}\right)}^{2}+{\left(\lambda_{3}-\lambda_{m}\right)}^{2}}{\lambda_{1}{}^{2}+\lambda_{2}{}^{2}+\lambda_{3}{}^{2}}}$$

MD: mean diffusivity; ADC: apparent diffusion coefficient; FA: fractional anisotropy; λ_1_: major eigenvalue; λ_2_ and λ_3_: minor eigenvalues; λ_m_: mean eigenvalue.

TraceW maps, MD maps, and FA maps were automatically generated using DTI processing software on the Siemens workstation (syngo MultiModality Workplace, version VE40B). Two observers with 9 years and 8 years of experience in pelvic MRI determined the maximum level of each testis from the MRI images and jointly determined the rules for plotting regions of interest. Regions of interest were outlined at the maximum level of the testes at b value = 0 s/mm^2^ along the outer border of the testes (right and left). These regions of interest were automatically replicated to the MD and FA parameter maps. The values of MD and FA were recorded.

### 2.4. Statistical Analysis

Continuous variables were summarized as mean (standard deviation) or median (interquartile ranges) and compared between groups with the Student’s *t*-test or Mann–Whitney *U* test when appropriate. Categorical variables were summarized as number (%). The intraclass correlation coefficient (ICC) was used to determine whether two observers were consistent in their measurement of data. Receiver operating characteristic (ROC) analysis was used to obtain the cutoff value of derived parameters of DTI and thereby determine the likelihood of sperm retrieval in NOA patients, and the area under the curve (AUC) as calculated to evaluate the diagnostic accuracy of the MD value. Statistical analyses were performed using SPSS 25.0 software (SPSS Inc., Chicago, IL, USA). Two-sided values of *p* < 0.05 were considered statistically significant.

## 3. Results

### 3.1. Characteristics of the Participants

**Figure 1:**
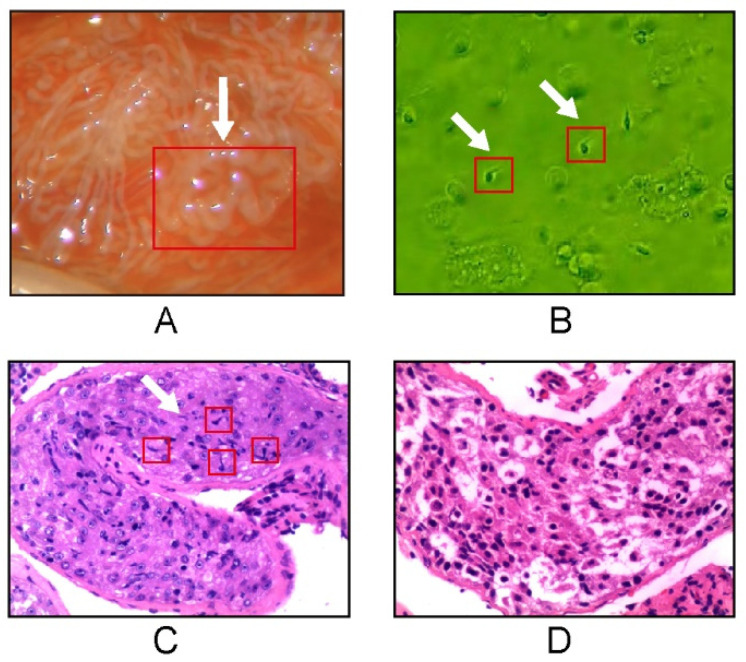
Histopathological examination. The mTESE shows that a milky white and thicker seminiferous tubule is selected (arrowhead) under an 18× microscope (**A**). After the selected milky white and thicker seminiferous tubule is cut up, mature sperm are found (arrowhead) under a 200× microscope (**B**). The Histopathology slide shows the seminiferous tubules of a NOA patient with sperm retrieval; few spermatozoa are distributed in the seminiferous tubule (arrowhead) (H&E, 200) (**C**). Histopathology slide shows the seminiferous tubules of a NOA patient without sperm retrieval; no mature sperm is found in the seminiferous tubule (H&E, 200) (**D**).

The success of sperm retrieval was confirmed according to the sperm obtained during the mTESE procedure. Representative examples and typical histopathology slides are shown in Figure 1. A total of 29 NOA patients with successful sperm retrieval (mean age, 30.2 years; age range, 24–40 years) and 41 NOA patients with failed sperm retrieval (mean age, 29.9 years; age range, 26–34 years) were prospectively collected. The duration of infertility for NOA patients who underwent successful sperm retrieval ranged from 1 to 18 years, with a mean infertility duration of 2.9 years. The duration of infertility for NOA patients with failed sperm retrieval ranged from 0.5 to 9 years, with a mean infertility duration of 2.9 years. Among the 29 NOA patients with successful sperm retrieval, 22 had unilateral mTESE, 7 had bilateral mTESE (sperm were retrieved from both testes), and a total of 36 testes were included for measurement. Among 41 NOA patients with failed sperm retrieval, 39 had bilateral mTESE, 2 had unilateral mTESE, and a total of 80 testes were included for measurement. The various clinical manifestations of the patients were as follows: idiopathic NOA (34 cases), 47XXY (14 cases), mumps/orchitis (13 cases), Y chromosome microdeletions of the AZFc (6 cases), reduction and fixation of cryptorchidism (2 cases) and treatment with radiotherapy (1 case) (Table 1). Sex hormones including follicle-stimulating hormone, luteinizing hormone, estradiol, testosterone, and prolactin, showed no statistically significant difference between successful and failed sperm retrieval in NOA patients (Table 2).

### 3.2. ICC Statistics between the Two Observers

The observer consistency test was analyzed, and the ICC of FA was 0.938 (*p* < 0.001), and the ICC of MD was 0.971 (*p* < 0.001), which indicated a high level of agreement between the two observers regarding the data measurements. Thus, we chose the data for analysis from one observer.

### 3.3. Comparisons in the Parameters between the Groups

**Figure 2:**
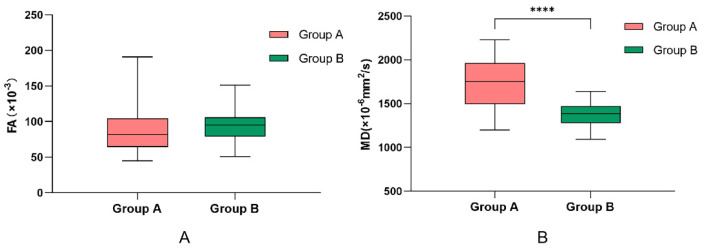
Boxplots show FA and MD distribution between the two groups. The mean FA value is not significantly different between the two groups (*p* > 0.05) (**A**). The mean MD value is significantly higher among the patients in Group A than among those in Group B (*p* < 0.001) (**B**). FA: fractional anisotropy, MD: mean diffusivity, ****: *p* < 0.001.

**Figure 3:**
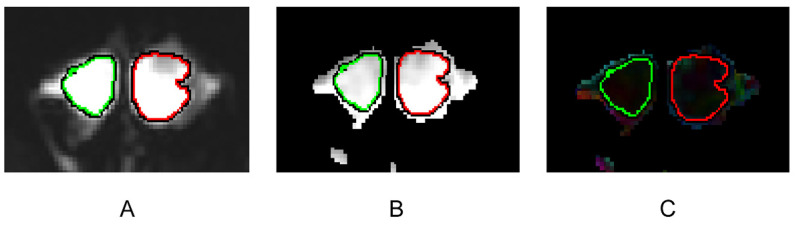
DTI parameter maps of a 34-year-old NOA patient with sperm retrieval. ROIs are outlined at the maximum level of the testes at b value = 0 s/mm^2^ along the outer border of the testes (right and left) (**A**). ROIs are automatically replicated to the MD (**B**) and FA (**C**) parameter maps. The MD values are 1974.7 × 10^−6^ mm^2^/s (left testis) and 1831.5 × 10^−6^ mm^2^/s (right testis). The FA values are 83.8 × 10^−3^ (left testis) and 68.1 × 10^−3^ (right testis).

**Figure 4:**
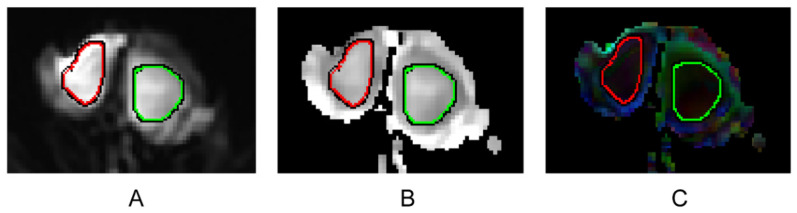
DTI parameter maps of a 30-year-old NOA patient without sperm retrieval. ROIs are outlined at the maximum level of the testes at b value = 0 s/mm^2^ along the outer border of the testes (right and left) (**A**). ROIs are automatically replicated to the MD (**B**) and FA (**C**) parameter maps. The MD values are 1355.6 × 10^−6^ mm^2^/s (left testis) and 1417 × 10^−6^ mm^2^/s (right testis). The FA values are 90.4 × 10^−3^ (left testis) and 125.9 × 10^−3^ (right testis).

The FA and MD values were compared between the 36 testes of the 29 NOA patients with successful sperm retrieval by mTESE (Group A) and the 80 testes of the 41 NOA patients with failed sperm retrieval by mTESE (Group B). The mean FA value was not significantly different between the two groups (*p* > 0.05). The mean MD value was significantly higher among the patients in Group A than among those in Group B (*p* < 0.001) (Table 3, Figure 2). Representative examples of the images of testes in Group A and Group B are shown in Figure 3 and Figure 4.

### 3.4. ROC Analysis

**Figure 5:**
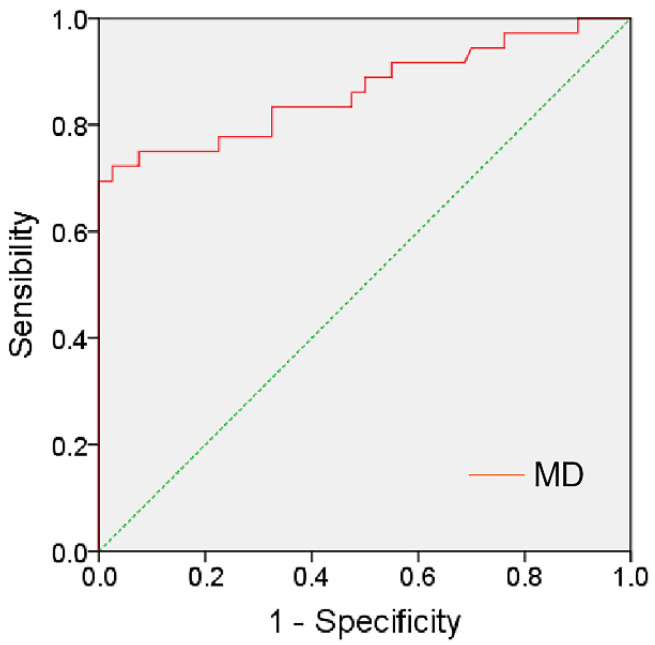
The ROC curves show the diagnostic accuracy of MD value in successful sperm retrieval of NOA patients.

The AUC with a 95% confidence interval for MD was 0.865 (0.780–0.950). The MD value showed a sensitivity of 72.2% and a specificity of 97.5% at the cutoff value of 1609.8 (Figure 5).

## 4. Discussion

This study evaluated the accuracy of quantitative parameter values of DTI in predicting the SRR, which might reveal the clinical applicability of these parameters.

Testicular MRI primarily uses T1-weighted and T2-weighted imaging for lesion diagnosis. However, the DTI sequence offers both qualitative and quantitative analysis by measuring water diffusion direction and extent, utilizing derived parameters like FA and MD.

The complexity of the testicular structure is the basis for the restricted diffusion of molecules. Recent studies have reported the favorable applicability of DTI in testicular research [17,18,25,29]. A recent study discovered that DTI could identify variations in ADC and FA values in the testicular microstructure of men with nonobstructive azoospermia in comparison to healthy controls. These differences were found to be significant [18]. Notably, FA values were useful for the diagnosis of testes in infertile men with varicoceles, with very good interobserver agreement [25]. Additionally, Tsili AC et al. demonstrated that both ADC and FA values significantly differed between testicular lesions and normal testes [17]. Moreover, Nissan N et al. established normative values for FA and MD in testicular parenchyma [29]. Currently, there is a lack of efficient and noninvasive methods for predicting SRR before mTESE. In our study, the MD values showed a high level of accuracy in predicting the SRR with a sensitivity of 72.2% and a specificity of 97.5%. Thus, MD values might be used as a noninvasive means of predicting SRR, avoiding unnecessary mTESE.

Spermatogonia in the seminiferous tubules of the testes is the starting point of sperm production. Via proliferation and meiosis, they give rise to all levels of spermatogenic cells leading to the formation of mature sperm. Additionally, the interstitial cells surrounding the spermatogonia are responsible for producing testosterone, which plays a role in promoting sperm production and development. A decrease or deletion in spermatogenic cells at any level leads to a decrease in tissue density and cellularity.

MD is a measure of the extent of water diffusion in tissue, regardless of the direction. It reflects the overall rate of water diffusion and is affected by tissue density and cellularity. MD values are usually reported in units of mm^2^/s and are inversely related to tissue density. This means that lower MD values indicate higher tissue density and higher cellularity.

Our results showed that MD values were significantly higher in Group A than in Group B (*p* < 0.001), with an AUC of 0.865, whereas Tsili AC et al. showed no significant difference in ADC values (equivalent to MD values) between the groups with and without sperm retrieval by mTESE [18]. The inconsistent results could be attributed to the varying proportions of NOA subtypes in these studies. Our finding showed significant differences in MD values between the successful and failed mTESE sperm retrieval groups. Potential explanations included a more severe testicular spermatogenic cell development disorder in the failed retrieval group and possible compensatory proliferation of testicular interstitial cells in the group without sperm retrieval, thus promoting androgen synthesis and sperm production. Additionally, in patients with NOA due to orchitis, those without sperm retrieval had a greater degree of spermatogenic cell damage and might have suffered a more severe inflammatory storm with more inflammatory cell infiltration. In addition, the chronic inflammatory process caused by more severe inflammatory stimulation might lead to increased proliferation or repair of fibrous connective tissue in the testicular interstitium. The NOA patients without sperm retrieved were more likely to generate more testicular interstitial cells, inflammatory cells, and fibrosis in the testis, leading to lower MD values compared to the NOA patients with sperm retrieved.

FA is a measure of the anisotropy or directionality of water diffusion in tissue. In biological tissue, water molecules tend to diffuse more easily along the directions of organized structures, such as axons in the brain or seminiferous tubules in the testis. The FA values range from 0 to 1, where 0 represents complete isotropy (random diffusion in all directions) and 1 represents complete anisotropy (diffusion in one direction only).

Our results showed FA values close to 0.1 in both Group A and Group B, which were similar to the results of Tsili AC et al. [18]. This indicated a small degree of testicular spread anisotropy in patients with NOA. In this study, there was no significant difference in FA values when comparing the sperm retrieval success group with the failure group in NOA patients. These findings aligned well with the results reported by Tsili AC et al. [18].

In recent years, a few studies have applied DTI to testicular disease research. A study by Tsili AC et al. showed significant differences in the FA values of testes with varicoceles compared to normal controls [25]. Another study by Tsili AC et al. revealed that compared to the testes of normal controls, the testes in the lesioned group had significantly different FA values [18]. These findings suggested that there was potential for effective diagnosis using FA values. Our study revealed no significant difference in FA values between the groups of successful and failed mTESE sperm retrieval. Similarly, in a study by Tsili AC et al. [18], no difference in the FA values of testes was found among different Johnsen score groups in patients with NOA. Furthermore, Tsili AC et al. conducted a study comparing FA values between testicular malignancy and benign nodules. The results showed no significant difference, suggesting that FA might not serve as a reliable marker for distinguishing between these two conditions [17]. In summary, recent studies found that FA values derived from DTI in testicular lesions and normal controls were significantly different. However, relying solely on FA values was not effective for accurately determining the grade and classification of testicular lesions. Further diagnostic tools or parameters were needed to improve the accuracy and reliability of testicular lesion classification.

Retrospective studies of the association between sex hormones and SRR were disputed [30]. Our study showed no statistically significant difference in sex hormones (follicle-stimulating hormone, luteinizing hormone, estradiol, testosterone, and prolactin) between the two groups. One possible explanation was that sex hormones are regulated by feedback from the hypothalamic-pituitary-testicular axis. Therefore, even in the same NOA patient, hormone levels were influenced by environmental factors and his own physical conditions, which led to instabilities in hormone levels. Due to the instabilities of sex hormones, it was difficult to use them as an efficient predictor of SSR in NOA patients.

Recent studies have shown that the overall success rate of mTESE was not high, and there was still a lack of noninvasive methods to predict the SRR. Although the implementation of DTI might significantly raise costs, the benefits of predicting the SRR using DTI were much higher than the high surgical costs, surgical trauma, and anesthesia risks caused by failed sperm retrieval. Siemens 3.0 T MRI devices were routinely equipped with image post-processing workstations, which allowed for automatic transmission and processing of images. The post-processing software could directly generate maps of MD parameters. Drawing the ROI on the MD parameter map and comparing the resulting values with the predefined cutoff values could be accomplished within approximately 5 min. In addition, the signal of the testis at b value = 0 s/mm^2^ was distinct from other tissues in the scrotum, with a clear boundary, making the ROI relatively easy to draw. Therefore, the image post-processing of DTI was feasible. As a result, there was a certain prospect for using DTI to predict the SRR in patients with NOA.

When using DTI to predict the SRR, there are various challenges and limitations to consider. Technical barriers exist as DTI is a complex imaging technique requiring high-quality equipment and software. Proper settings and skilled operators are essential for accurate results. Image quality can be affected by motion artifacts, susceptibility artifacts, and distortion, potentially impacting prediction accuracy. Clinical variability, including biological and anatomical differences, must be accounted for in establishing accurate baseline data. However, collecting a sufficient sample size is challenging due to the need for diverse and representative clinical data. Predicting SRR is complex and involves analyzing multiple factors, such as testicular pathology, patient age, and hormone levels. A comprehensive model is necessary to improve prediction accuracy by considering interactions and trade-offs among these factors. In conclusion, while DTI holds promise for predicting SRR, addressing technical and clinical challenges is crucial for its widespread use in clinical practice.

Our study also had some limitations that should be considered. First, our study was a single-center investigation with a relatively insufficient sample size. The automated classification and segmentation of urinary multiparametric MRI images using convolutional neural networks have demonstrated high diagnostic accuracy [31,32]. Future prospective multicenter studies with large samples are needed to validate the diagnostic accuracy of an automated approach using convolutional neural networks in testicular multiparametric MRI. Second, further investigation is needed to determine whether the FA value can predict the success rate of sperm retrieval in patients with NOA in a noninvasive manner. This investigation should include the use of different MRI devices, varying numbers of diffusion gradient directions, as well as different b values. Finally, exploring the use of DTI in combination with other imaging techniques is needed to provide more comprehensive and specific recommendations for future research.

## 5. Conclusions

MD values were significantly lower in the group without sperm retrieval than in the group with sperm retrieval. As an important parameter of DTI, MD might be used as a noninvasive imaging marker for predicting the SRR in patients with NOA.

## Figures and Tables

**Table 1:** Basic characteristics of study participants by groups whether sperm is retrieved or not.

Parameters	Total Patients (*n* = 70)	Patients with Successful Sperm Retrieval (*n* = 29)	Patients with Failed Sperm Retrieval (*n* = 41)
Age, years	30 (24–40)	30.2 (24–40)	29.9 (26–34)
Duration of infertility, years	2.9 (0.5–18)	2.9 (1–18)	2.9 (0.5–9)
Testes included, *n*	116 (24 unilateral, 46 bilateral)	36 (22 unilateral, 7 bilateral)	80 (2 unilateral, 39 bilateral)
Clinical manifestations, *n*			
Idiopathic NOA	34	12	22
47XXY	14	7	7
Mumps/orchitis	13	7	6
Y chromosome microdeletions of the AZFc	6	2	4
Reduction and fixation of cryptorchidism	2	1	1
Treatment with radiotherapy	1	0	1

Data are expressed as *n* for categorical data and mean (range) for parametrically distributed data. Abbreviations: NOA, nonobstructive azoospermia.

**Table 2:** The differences in sex hormones between the two groups.

Parameters	Patients with Successful Sperm Retrieval (*n* = 29)	Patients with Failed Sperm Retrieval (*n* = 41)	T/Z	*p* Value
Follicle-stimulating hormone, mIU/mL	32.48 ± 18.18	26.31 ± 14.61	−1.571	0.121 ^a^
Luteinizing hormone, mIU/mL	11.73 (6.92–18.90)	8.31 (6.15–13.13)	1.404	0.085 ^b^
Estradiol, pg/mL	24.19 ± 10.86	25.80 ± 13.99	0.492	0.624 ^a^
Testosterone, ng/dL	298.65 ± 169.03	339.64 ± 186.47	0.941	0.350 ^a^
Prolactin, ng/mL	10.30 (8.58–15.77)	9.72 (7.80–13.53)	1.772	0.520 ^b^

Data are presented as mean ± standard deviation or median (interquartile range) as appropriate. ^a^ Comparisons are performed by the Student’s *t*-test. ^b^ Comparisons are performed by the Mann–Whitney *U* test.

**Table 3:** Comparison of the fractional anisotropy and mean diffusivity values between the two groups.

Parameters	Group A (*n* = 36)	Group B (*n* = 80)	T	*p* Value ^a^
Fractional anisotropy, ×10^−3^	88.8 ± 33.4	94.0 ± 21.3	1.131	0.293
Mean diffusivity, ×10^−6^ mm^2^/s	1717.6 ± 271.5	1378.1 ± 129.9	−8.959	0.000

Data are presented as mean ± standard deviation as appropriate. Fractional anisotropy is non-dimensional. Group A consisted of 36 testes of the 29 NOA patients with successful sperm retrieval; Group B consisted of 80 testes of the 41 NOA patients with failed sperm retrieval. ^a^ Comparisons are performed by the Student’s *t*-test.

## Data Availability

Data underlying the results presented in this paper are not publicly available at this time but may be obtained from the authors upon reasonable request.
